# Supplementary material for: Lamina-specific cortical dynamics in human visual and sensorimotor cortices
Source: eLife. 2018 Oct 22;7:e33977. doi: 10.7554/eLife.33977 (PMC6197856; doi:10.7554/eLife.33977)
Supplement: Transparent reporting form [file elife-33977-transrepform.docx]

***eLife’s* transparent reporting form**

We encourage authors to provide detailed information *within their submission* to facilitate the interpretation and replication of experiments. Authors can upload supporting documentation to indicate the use of appropriate reporting guidelines for health-related research (see [EQUATOR Network](http://www.equator-network.org/%20)), life science research (see the [BioSharing Information Resource](https://biosharing.org/)), or the [ARRIVE guidelines](http://www.plosbiology.org/article/info:doi/10.1371/journal.pbio.1000412) for reporting work involving animal research. Where applicable, authors should refer to any relevant reporting standards documents in this form.

If you have any questions, please consult our Journal Policies and/or contact us: [editorial@elifesciences.org](mailto:editorial@elifesciences.org).

**Sample-size estimation**

- You should state whether an appropriate sample size was computed when the study was being designed
- You should state the statistical method of sample size computation and any required assumptions
- If no explicit power analysis was used, you should describe how you decided what sample (replicate) size (number) to use

Please outline where this information can be found within the submission (e.g., sections or figure legends), or explain why this information doesn’t apply to your submission:

This was an exploratory study as it was using cutting edge high precision MEG techniques which have not previously been used to investigate laminar specificity. Our previous simulation work has shown that given sufficient SNR, it is possible to discriminate between source activity in deep versus superficial cortical laminae (Troebinger et al., 2014; Bonaiuto et al., 2017). However, because laminar analyses have not previously been attempted with human MEG data, we chose to maximize the number of trials per participant, based on their tolerance. We therefore tested eight participants, with 540-2700 trials per participant, split into 3 separate runs, over 1-5 days. For these reasons, no formal power calculations were possible.

The laminar analyses are performed on each participant separately as a series of case studies, rather than at a group level, and figures 5-6 show raw data for a representative participant as well as the analysis results for each participant. The task condition modulation analyses are performed at a group level using nonparametric tests, and figures 7-8 show a representative participant as well as the normalized group level and individual participant values.

**Replicates**

- You should report how often each experiment was performed
- You should include a definition of biological versus technical replication
- The data obtained should be provided and sufficient information should be provided to indicate the number of independent biological and/or technical replicates
- If you encountered any outliers, you should describe how these were handled
- Criteria for exclusion/inclusion of data should be clearly stated
- High-throughput sequence data should be uploaded before submission, with a private link for reviewers provided (these are available from both GEO and ArrayExpress)

Please outline where this information can be found within the submission (e.g., sections or figure legends), or explain why this information doesn’t apply to your submission:

N/A – we performed the experiment once

**Statistical reporting**

- Statistical analysis methods should be described and justified
- Raw data should be presented in figures whenever informative to do so (typically when N per group is less than 10)
- For each experiment, you should identify the statistical tests used, exact values of N, definitions of center, methods of multiple test correction, and dispersion and precision measures (e.g., mean, median, SD, SEM, confidence intervals; and, for the major substantive results, a measure of effect size (e.g., Pearson's r, Cohen's d)
- Report exact p-values wherever possible alongside the summary statistics and 95% confidence intervals. These should be reported for all key questions and not only when the p-value is less than 0.05.

Please outline where this information can be found within the submission (e.g., sections or figure legends), or explain why this information doesn’t apply to your submission:

The analysis methods are described in the Materials and Methods section.

(For large datasets, or papers with a very large number of statistical tests, you may upload a single table file with tests, Ns, etc., with reference to sections in the manuscript.)

**Group allocation**

- Indicate how samples were allocated into experimental groups (in the case of clinical studies, please specify allocation to treatment method); if randomization was used, please also state if restricted randomization was applied
- Indicate if masking was used during group allocation, data collection and/or data analysis

Please outline where this information can be found within the submission (e.g., sections or figure legends), or explain why this information doesn’t apply to your submission:

N/A – this study reports a series of single case studies, and summarizes some of the results in a single group

**Additional data files (“source data”)**

- We encourage you to upload relevant additional data files, such as numerical data that are represented as a graph in a figure, or as a summary table
- Where provided, these should be in the most useful format, and they can be uploaded as “Source data” files linked to a main figure or table
- Include model definition files including the full list of parameters used
- Include code used for data analysis (e.g., R, MatLab)
- Avoid stating that data files are “available upon request”

Please indicate the figures or tables for which source data files have been provided:

All data are archived at the Open MEG Archive (OMEGA; <http://dx.doi.org/10.23686/0015896>). All data analysis code is available on github (<https://github.com/jbonaiuto/meg-laminar>). Numerical data for figures 1-3, 5-8 are included as source data files.
